# Integrated single-cell and bulk transcriptomics identify autophagy-related immune-suppressive subtypes and a prognostic signature in colorectal cancer

**DOI:** 10.3389/fimmu.2026.1820929

**Published:** 2026-04-21

**Authors:** Yueming Wu, Lan Teng, Lihua Zhou, Side Liu, Yubin Guo

**Affiliations:** Guangdong Provincial Key Laboratory of Gastroenterology, Department of Gastroenterology, Nanfang Hospital, Southern Medical University, Guangzhou, Guangdong, China

**Keywords:** autophagy, colorectal cancer, GOLGA2, machine learning, single-cell RNA sequencing, tumor microenvironment

## Abstract

**Background:**

Colorectal cancer still causes many cancer deaths, and patient outcomes differ a lot. The tumor microenvironment can shape tumor growth and treatment response. Autophagy is a cell recycling process linked to tumor survival and immune control, but its cell-type pattern in colorectal cancer tissue and its clinical meaning are not clear. We mapped autophagy activity across cell types, defined autophagy-based subtypes, and tested their value for risk stratification.

**Methods:**

Single-cell RNA sequencing data from colorectal cancer tumor tissues were integrated to evaluate autophagy activity across malignant, stromal, and immune cell populations. Subsequently, autophagy-related genes were curated from multiple public databases and analyzed in bulk transcriptomic cohorts obtained from The Cancer Genome Atlas and The Gene Expression Omnibus. Additionally, unsupervised consensus clustering was applied to define autophagy-based molecular subtypes. Multiple machine learning algorithms were evaluated to construct an autophagy-related prognostic model, and feature importance was assessed using explainable modeling approaches. Furthermore, immune microenvironment characteristics, genomic alterations, and stemness features were systematically analyzed. Key genes were further validated in clinical colorectal cancer specimens and through *in vitro* functional experiments.

**Results:**

Autophagy activity varied across the tumor microenvironment. Malignant and stromal cells showed higher autophagy levels than immune cells. High-autophagy tumors showed stronger tumor–stroma interactions, weaker immune communication, and an immune-suppressive microenvironment. Using 47 prognostic autophagy-related genes, we identified four subtypes with different overall survival, genomic alteration patterns, stemness signatures, and immune landscapes. The prognostic model performed well across independent cohorts. GOLGA2 (Golgi autoantigen, golgin subfamily A member 2) was the top risk gene and was upregulated in tumor tissues. Functional assays showed that GOLGA2 promotes HCT116 cell proliferation and migration.

**Conclusion:**

Autophagy is closely tied to an immune-suppressive tumor microenvironment in colorectal cancer. The autophagy-based subtypes and prognostic model support patient risk stratification. Furthermore, GOLGA2 was a top-ranked gene in the signature and showed tumor-promoting effects *in vitro*, so it may warrant further study as a potential target.

## 1 Introduction

Colorectal cancer (CRC) is the third most frequently diagnosed malignancy and the second leading cause of cancer-related mortality worldwide (1). Despite advances in surgical techniques, systemic chemotherapy, and immunotherapy, clinical outcomes remain highly heterogeneous, largely due to therapy resistance, metastatic progression, and disease recurrence (2). Increasing evidence indicates that these challenges are not solely determined by tumor-intrinsic factors but are profoundly influenced by the tumor microenvironment (TME) (3).

The CRC TME is characterized by extensive stromal remodeling and immune dysregulation. Stromal components, including cancer-associated fibroblasts and endothelial cells, together with immunosuppressive populations such as regulatory T cells and M2-type tumor-associated macrophages, collectively limit effective antitumor immune responses and reduce therapeutic efficacy (4–6). Moreover, anticancer therapies themselves can reshape the TME, promoting the survival of stress-adapted tumor and stromal cells and driving a transition toward an immune-suppressive state (7, 8). However, the molecular mechanisms that coordinate these adaptive responses within distinct TME compartments remain incompletely understood.

Autophagy, a conserved lysosomal degradation pathway, plays a central role in cellular adaptation to metabolic, oxidative, and therapeutic stress (9–11). In established tumors, autophagy supports cancer cell survival under hypoxia and nutrient deprivation, contributes to therapeutic resistance, and promotes tumor progression (12–14). Importantly, accumulating evidence suggests that autophagy also regulates the tumor immune microenvironment by modulating antigen presentation, T-cell function, macrophage polarization, and stromal remodeling (15–17). These functions position autophagy as a potential integrator of tumor–stroma–immune interactions in CRC.

However, current evidence does not explain how autophagy differs across malignant cells, stromal cells, and immune cells in the CRC TME. It is also unclear which cell compartments contribute most to an immune-suppressive or immune-excluded state. This gap matters because CRC treatment response depends on cell–cell interactions in the microenvironment, not only on tumor cells. We therefore asked whether cell-type autophagy heterogeneity can define reproducible microenvironment patterns, and whether these patterns are linked to patient outcome and immune features.

To address this gap, we integrated single-cell RNA sequencing with bulk transcriptomic cohorts to map autophagy activity across major TME compartments. We then defined autophagy-related molecular subtypes and evaluated their associations with genomic features, stemness-related signals, immune landscapes, and survival. We also built an autophagy-related prognostic model using machine learning and tested its performance in independent cohorts. Finally, we validated GOLGA2 in clinical tissues and *in vitro* assays to connect the computational results with functional evidence.

## 2 Materials and methods

### 2.1 Data collection

Single-cell RNA sequencing (scRNA-seq) data of colorectal cancer were retrieved from the TISCH database (https://tisch.compbio.cn/), including the datasets CRC_EMTAB8107, CRC_GSE146771_Smart-seq2, and CRC_GSE139555. This project analyzed 20 CRC samples Bulk RNA-seq data were obtained from the UCSC Xena and GEO databases, including TCGA-COAD (n = 471), TCGA-READ (n = 166), GSE39582 (n = 566), and GSE17538 (n = 238).

### 2.2 Single-cell differential expression and pathway analysis

Single-cell RNA sequencing data were processed using the Seurat R package. Cell filtering, normalization, clustering, and annotation were performed based on the annotations provided by the TISCH database. Batch effects across samples were corrected using Harmony in PCA space, and sample mixing within major cell types was checked by Uniform Manifold Approximation and Projection (UMAP) visualization after correction. Cells were clustered using a shared nearest neighbor graph and Louvain community detection. The clustering resolution was selected by comparing multiple resolutions and evaluating cluster stability and biological interpretability based on known marker genes. Differentially expressed genes (DEGs) were identified using the FindAllMarkers function with thresholds of |log_2_ fold change| ≥ 1, false discovery rate (FDR) < 0.05 and min.pct = 0.25.

Pathway activity was assessed by Gene Set Variation Analysis (GSVA). To reduce the impact of sparsity and technical noise in single-cell data, GSVA scores were calculated at the cluster level using aggregated expression profiles. The resulting pathway scores were used for between-group comparisons and functional interpretation. The TISCH2 online platform was used to evaluate the cell type-specific expression pattern of GOLGA2 in CRC single-cell datasets.

### 2.3 Bulk RNA-seq data preprocessing, normalization, and cohort integration

TCGA RNA-seq and GEO microarray cohorts were processed separately because they were generated on different platforms. For TCGA RNA-seq, the gene-level expression matrix was retained, and log2(x+1) transformation was applied when the value distribution was high. No cross-platform normalization was applied to preserve RNA-seq characteristics. For GEO microarray cohorts, preprocessing was performed using the limma framework. Probes were mapped to gene symbols, duplicated genes were collapsed using avereps, quantile normalization was performed using normalizeBetweenArrays, and expression values were kept on the log2 scale.

To construct the merged cohort, the intersection of genes shared across TCGA, GSE17538, and GSE39582 was extracted and aligned into a unified gene expression matrix. Batch effects across data sources were corrected using ComBat (sva package) with empirical Bayes adjustment, with dataset source defined as the batch variable.2.3 Autophagy scores and Autophagy-Related Genes.

Autophagy scores were calculated for each single cell using the GSVA R package (method = “zscore”). The input gene set was the MSigDB Hallmark autophagy gene set (v2023.2). Autophagy-related genes (ARGs) were collected from the Molecular Signatures Database (MSigDB) database, including KEGG_REGULATION_OF_AUTOPHAGY, REACTOME_AUTOPHAGY, WP_AUTOPHAGY, and GOBP_REGULATION_OF_AUTOPHAGY gene sets.

### 2.4 Machine-learning model construction and validation

TCGA was used as the training cohort. GSE17538 and GSE39582 were used as independent external validation cohorts. A merged cohort was generated by combining TCGA, GSE17538, and GSE39582 for overall evaluation only for overall evaluation and visualization, and it was not used for feature selection, parameter tuning, or model training.

All feature selection, parameter tuning, model fitting, and internal cross-validation were performed only in TCGA, and external cohorts were not used for feature screening or optimization. Ten survival models were evaluated (RSF, LASSO, GBM, Survival-SVM, SuperPC, Ridge, plsRcox, CoxBoost, Stepwise Cox, and Elastic Net), and cross-cohort performance was compared using C-index and mean C-index. LASSO and Elastic Net used 10-fold cross-validation to select the optimal λ (Elastic Net used an α grid from 0.1 to 0.9). CoxBoost used K-fold cross-validation to select the optimal boosting steps. GBM used shrinkage = 0.001 and interaction depth = 3, and selected the number of trees by cross-validation error. plsRcox selected the number of components by cross-validation. SuperPC used 10-fold cross-validation to select thresholds and components. The final RSF model used ntree = 1000, nodesize = 5, and splitrule = log-rank.

After training, the finalized model with fixed parameters was applied to the external cohorts and the merged cohort to calculate risk scores. Model performance was assessed using C-index, time-dependent ROC, and Kaplan–Meier analysis with log-rank test.

### 2.5 Clinical specimens

This study was approved by the Ethics Committee of Nanfang Hospital (Approval No. NFEC-2023-109). Human colorectal cancer tissues were obtained from patients undergoing surgical resectionafter written informed consent was obtained. A diagnosis of CRC was confirmed histopathologically for each sample and none of these patients had received chemotherapy or radiotherapy before surgery.

### 2.6 Cell culture

Human CRC HCT116 cell lines were purchased from the Cell Bank of Type Culture Collection (CBTCC, China Academy of Sciences, Shanghai, China). All cells were cultured in Dulbecco’s modified Eagle’s medium (DMEM) (Gibco, Carlsbad, CA) supplemented with 10% fetal bovine serum (FBS; Gibco, Carlsbad, CA) at 37 °C with a humidity of 5% CO2.

### 2.7 Gene transfection

GOLGA2-specific siRNAs and negative control siRNA were purchased from Sangon Biotech (**Supplementary Table 1**). A GOLGA2 overexpression plasmid was transiently transfected using Lipofectamine 3000 according to the manufacturer’s protocol.

### 2.8 Cell proliferation assay

Cell proliferation was estimated using a Resazurin Cell Viability Kit (Cell Signaling, catalog no. 11884). Cell viability was quantified by fluorescence after cellular dehydrogenases reduced resazurin to resorufin (excitation:530 nm-570 nm; emission:590 nm-620 nm). HCT116 cells were seeded into 96-well plates (2,000 cells/well). Each day, the culture medium was replaced with fresh medium containing 10% resazurin solution and incubated for 2 h before measurement. Fluorescence was detected at 530/590 nm daily for three consecutive days. Wells containing media and resazurin but without cells were used as blanks.

### 2.9 RNA extraction and quantitative RT-PCR

Total RNA was extracted from tissues and cells using TRIzol reagent (TaKaRa, Japan). cDNA was synthesized using the Hifair III 1st Strand cDNA Synthesis SuperMix (Yeasen, Shanghai, China), and quantitative PCR was performed using SYBR Green Premix Pro Taq HS (Accurate Biology, Changsha, China). Relative mRNA expression levels were calculated using the 2^-∆∆CT method, normalized to control genes. Primer sequences are listed in **Supplementary Table 2**.

### 2.10 Western blotting and immunohistochemistry

For Western blot analysis, protein samples were separated by 10% SDS-PAGE and transferred to PVDF membranes. Membranes were incubated with anti-GOLGA2 (1:1,000, Proteintech, 82705-8-RR) and anti-β-actin (1:10,000, Proteintech, 20536-1-AP) antibodies, followed by incubation with HRP-conjugated secondary antibody (1:1,000, Beyotime, Shanghai). Signals were visualized using an imaging system.

IHC staining was performed using an IHC kit (PV-6001, ZSGB-BIO, Beijing) according to the manufacturer’s instructions. Anti-GOLGA2 antibody (1:500, Proteintech) was used as the primary antibody.

### 2.11 Wound healing and migration assays

For wound healing assays, 4.5 × 10⁵ HCT116 cells were seeded into 6-well plates. At approximately 80% confluence, a scratch was made using a sterile pipette tip. Wound closure was monitored and imaged under a microscope.

Transwell migration assays were performed using 24-well inserts (8 μm pores, Corning). The lower chamber was filled with 600 μL of 10% FBS medium. A total of 3 × 10^4^ HCT116 cells in 200 μL of serum-free medium were gently loaded onto each filter insert (upper chamber) and then incubated for 48h. The filter inserts were removed from the chambers, fixed with 4% paraformaldehyde for 10min. A 0.5% crystal violet staining solution in methanol was diluted 1:5 with phosphate buffer saline (PBS) and used to stain the samples for 15 min. The samples were subsequently washed, dried and mounted onto slides. The migratory cells were stained purple, visualized under and inverted microscope and then counted in five random fields for statistical analysis. Each experiment was performed in triplicate.

### 2.12 Drug sensitivity prediction

Drug sensitivity was predicted using the oncoPredict R package. The training expression matrix was the GDSC2 expression dataset (RMA normalized and log-transformed), and the training drug response matrix was the GDSC2 drug response dataset. Predicted median inhibition concentration (IC50) values were compared between groups for each compound using a two-sided unpaired Student’s t-test. Compounds were ranked by P value, and the top 9 compounds with the smallest P values were visualized.

### 2.13 Statistical analysis

Statistical analyses were performed using R software 4.2.2, GraphPad Prism 8, and Fiji ImageJ v1.54r. Data are presented as mean ± SD. Differences between groups were analyzed using Student’s t-test. Matched tumor and adjacent tissues in immunohistochemistry were analyzed using paired Student’s t-test. Survival analyses were conducted using Kaplan–Meier curves with log-rank tests. A p-value < 0.05 was considered statistically significant.

## 3 Results

### 3.1 Autophagy enhances tumor-stroma interactions and suppresses anti-tumor immunity in the TME of CRC

**Figure 1:**
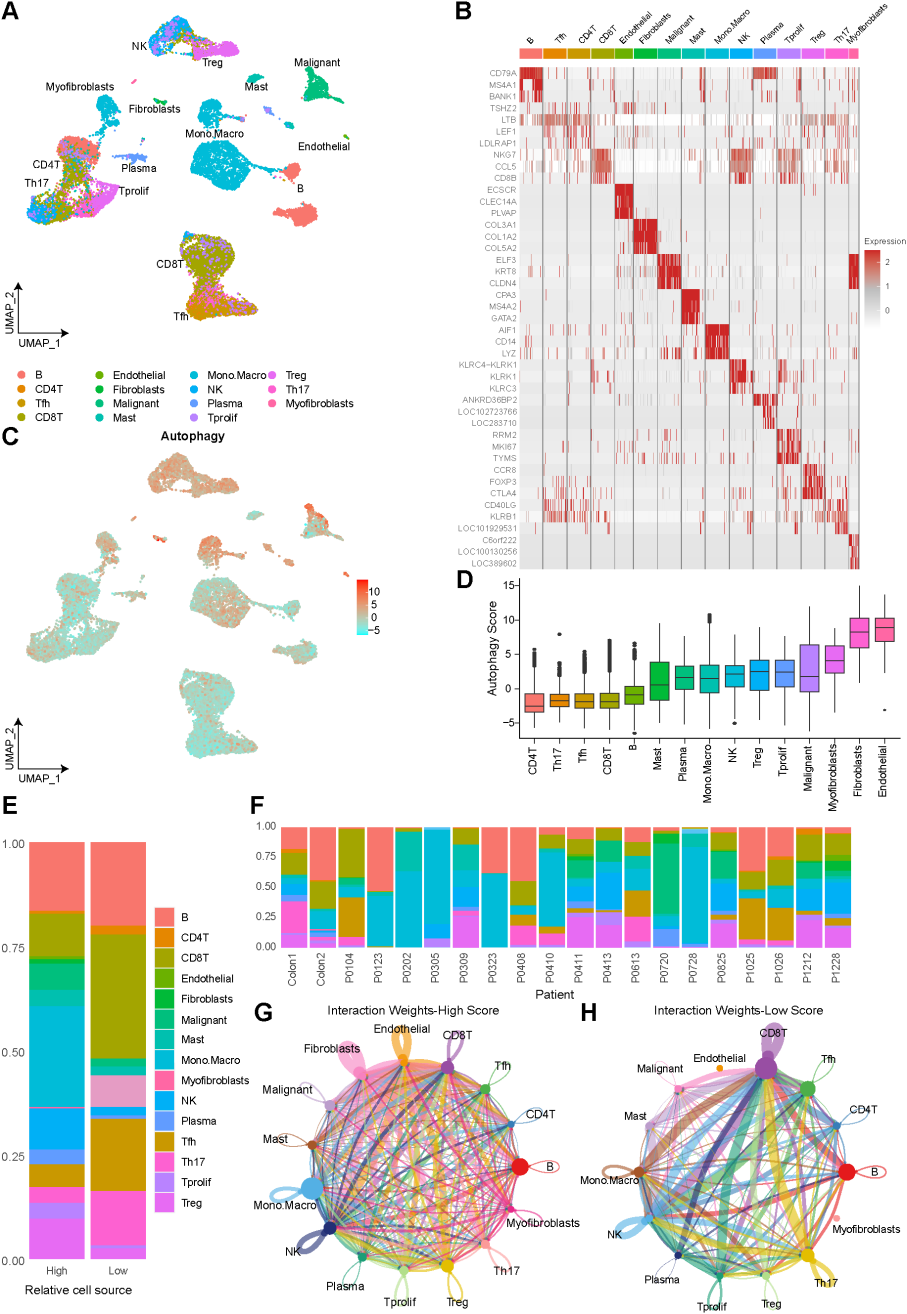
Single-cell landscape of autophagy activity and cell–cell communication in colorectal cancer. **(A)** UMAP visualization of 15 major cell types identified by single-cell RNA sequencing in CRC; **(B)** Heatmap showing the expression of the top three cell-type-specific marker genes derived from COSG analysis; **(C)** UMAP projection of cells stratified into high- and low-autophagy groups based on the median autophagy score; **(D)** Autophagy activity scores computed by GSVA for each cell; **(E)** Proportional distribution of tumor–stromal and immune cell populations in the high- and low-autophagy groups; **(F)** Proportion of each cell cluster across individual patients; **(G, H)** Weight of cellular interactions in the high-autophagy **(G)** and low-autophagy **(H)** groups.

The single-cell RNA sequencing (scRNA-seq) data from colorectal cancer (CRC) tissues were obtained from three Gene Expression Omnibus (GEO) datasets (GSE139555, GSE146771_10X, GSE146771_Smartseq2) and analyzed using the Tumor Immune Single-cell Hub (TISCH) database (https://tisch.compbio.cn/). After merging the datasets and correcting for batch effects, resulting in a final integrated dataset consisting of 22,816 cells derived from tumor tissues, representing expression profiles for 18,424 genes (**Supplementary Figure 1**). Following dimensionality reduction and clustering, the dataset revealed 12 distinct cell clusters (**Figure 1A**). These clusters were grouped into four major categories: lymphocytes (B, CD4T, Tfh, CD8T, Tprolif, Treg, Th17, Plasma, NK), myeloid cells (Monocyte/Macrophage, Mast), stromal cells (Endothelial, Fibroblasts, Myofibroblasts), and malignant epithelial cells. The top five marker genes for each cluster, as identified by COSG analysis, were visualized in a heatmap (**Figure 1B**), confirming the validity of the clustering and dimensionality reduction approach. Additionally, hallmark pathway analysis of the top 100 expressed genes per cell type revealed consistent upregulation of pathways related to essential cellular processes, metabolism, stress and damage response, proliferation, cell cycle regulation, and signal transduction in myeloid, stromal, and malignant cells (**Supplementary Figure 2A**).

Autophagy activity scores were calculated at the single-cell level using GSVA (**Figure 1C**). High-autophagy cells were predominantly found in malignant, stromal, and myeloid lineages, while low-autophagy cells were primarily associated with lymphoid clusters. Notably, autophagy scores were significantly higher in endothelial cells, fibroblasts, myofibroblasts, and malignant cells compared to lymphocytes (including CD4⁺ T, CD8⁺ T, and B cells) (**Figure 1D**). Based on median autophagy scores, cells were classified into high- and low-autophagy groups. Proportional analysis showed that the low-autophagy group was largely composed of lymphocytes (88.36%), a hallmark of strong anti-tumor immunity and favorable immunotherapy response (**Figure 1E**). In contrast, the high-autophagy group exhibited a significantly higher proportion of monocyte/macrophage cells (24.17% vs. 7.6%) and a marked reduction in CD8⁺ T cells. The enrichment of myeloid cells (especially monocyte/macrophage cells) and the increased presence of malignant and stromal cells in the high-autophagy group indicated a shift toward an immunosuppressive and tumor-promoting microenvironment. Moreover, the cellular composition varied substantially across individual patients. (**Figure 1F**).

Furthermore, pathway enrichment analysis indicated significant activation of cellular processes, metabolic pathways, stress responses, proliferation and cycle regulation, and signal transduction pathways in the high-autophagy group (**Supplementary Figure 2B**). In terms of cellular crosstalk, the high-autophagy group showed significantly higher interaction numbers and interaction strength compared to the low-autophagy group (interaction weights: 403.759 vs. 71.65, P < 0.001; **Figures 1G, H**). Notably, communication between tumor and stromal cells (such as pro-angiogenic and stromal remodeling signals like VEGF and TGF-β) was enhanced under high-autophagy conditions, whereas immune cell interactions (such as MHC antigen presentation and co-stimulatory signaling) were significantly suppressed (**Supplementary Figure 3**).

Collectively, these findings suggest that autophagy promotes the pro-tumorigenic properties of the TME by enhancing tumor-stroma communication while suppressing immune cell interactions.

### 3.2 Autophagy-related genes integration identifies four distinct molecular subtypes of CRC

**Figure 2:**
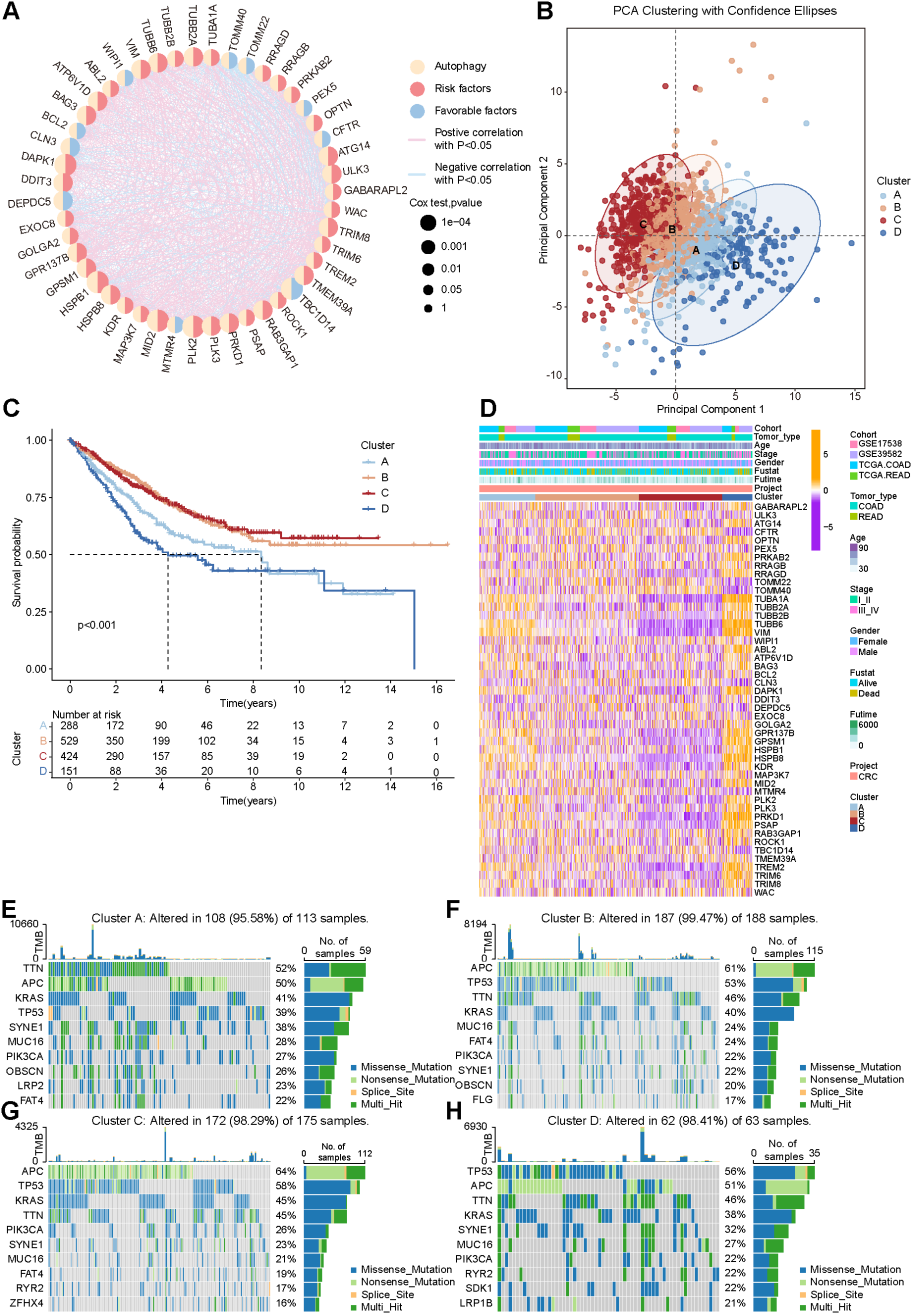
Identification of autophagy-related molecular subtypes in colorectal cancer. **(A)** Correlation analysis and univariate Cox regression of autophagy-related genes (ARGs) in the TCGA and GEO cohorts (GSE39582 and GSE17538). The right semicircle indicates prognostic risk (purple) or protective (green) effects, with circle size representing P values. Connecting lines denote significant correlations (P < 0.05). 47 ARGs with Cox P < 0.01 were selected for downstream analysis; **(B)** Unsupervised consensus clustering based on the 47 ARGs, identifying four optimal molecular subtypes (Clusters **A–D**), with principal component analysis (PCA) visualization; **(C)** Kaplan–Meier overall survival analysis of the four autophagy-related subtypes (P < 0.001); **(D)** Heatmap showing expression patterns of differentially expressed ARGs across the four subtypes; **(E–H)** Mutational landscape of the four subtypes **(A–D)**.

To further investigate the role of autophagy in CRC, autophagy-related genes set from KEGG, Reactome, WikiPathways, and Gene Ontology (KEGG_REGULATION_OF_AUTOPHAGY, REACTOME_AUTOPHAGY, WP_AUTOPHAGY, and GOBP_REGULATION_OF_AUTOPHAGY) were integrated. After merging and removing duplicates, 499 unique autophagy-related genes (ARGs) were obtained, of which 459 were expressed in the transcriptomic datasets used in this study. Correlation analysis and univariate Cox regression performed across 1,441 CRC samples derived from TCGA and GEO datasets identified 47 ARGs significantly associated with overall survival (Cox P < 0.01; **Figure 2A**). Based on the expression profiles of these 47 ARGs, unsupervised consensus clustering identified four distinct molecular subtypes of CRC (Clusters A–D), representing the optimal clustering solution (**Figure 2B**). Kaplan–Meier analysis revealed significant differences in overall survival among the four subtypes (P < 0.001; **Figure 2C**). Notably, Cluster D exhibited the poorest overall survival, whereas Cluster C showed the most favorable prognosis. Patients in Clusters B and C exhibited higher survival rates compared with those in Clusters A and D.

Differential expression analysis identified 41 ARGs with significantly differential expression among the four clusters (P < 0.001; **Figure 2D**). Mutational profiling revealed a high prevalence of somatic mutations across all clusters. Clusters B and C displayed comparable mutation frequencies in APC and TP53, while Clusters A and D exhibited a reduced APC mutation frequency of approximately 50% (**Figures 2E–H**). Distinct patterns of mutational heterogeneity were observed among the clusters, further supporting subtype-specific genomic characteristics (**Supplementary Figure 4A**). Stemness analysis showed that Cluster D exhibited significantly higher enrichment scores for hematopoietic and mesenchymal stem cell signatures, indicating enhanced stemness properties (**Supplementary Figure 4B**).

To further characterize the TME, immune cell infiltration was quantified using seven computational algorithms, including MCP-counter, EPIC, CIBERSORT, IPS, quanTIseq, ESTIMATE, and TIMER. Compared with Clusters B and C, Cluster D showed significantly increased infiltration of stromal cells, endothelial cells, and M2 macrophages, along with elevated overall immune and stromal scores, consistent with an immune-suppressive estimated phenotype. For predicted immunotherapy response, Cluster C demonstrated higher Immunophenoscore (IPS) values than Clusters A and D (**Supplementary Figure 5**). Collectively, these results define four autophagy-related molecular subtypes of CRC with distinct transcriptomic, genomic, stemness, and immune features.

### 3.3 Construction and validation of an autophagy-related prognostic model

**Figure 3:**
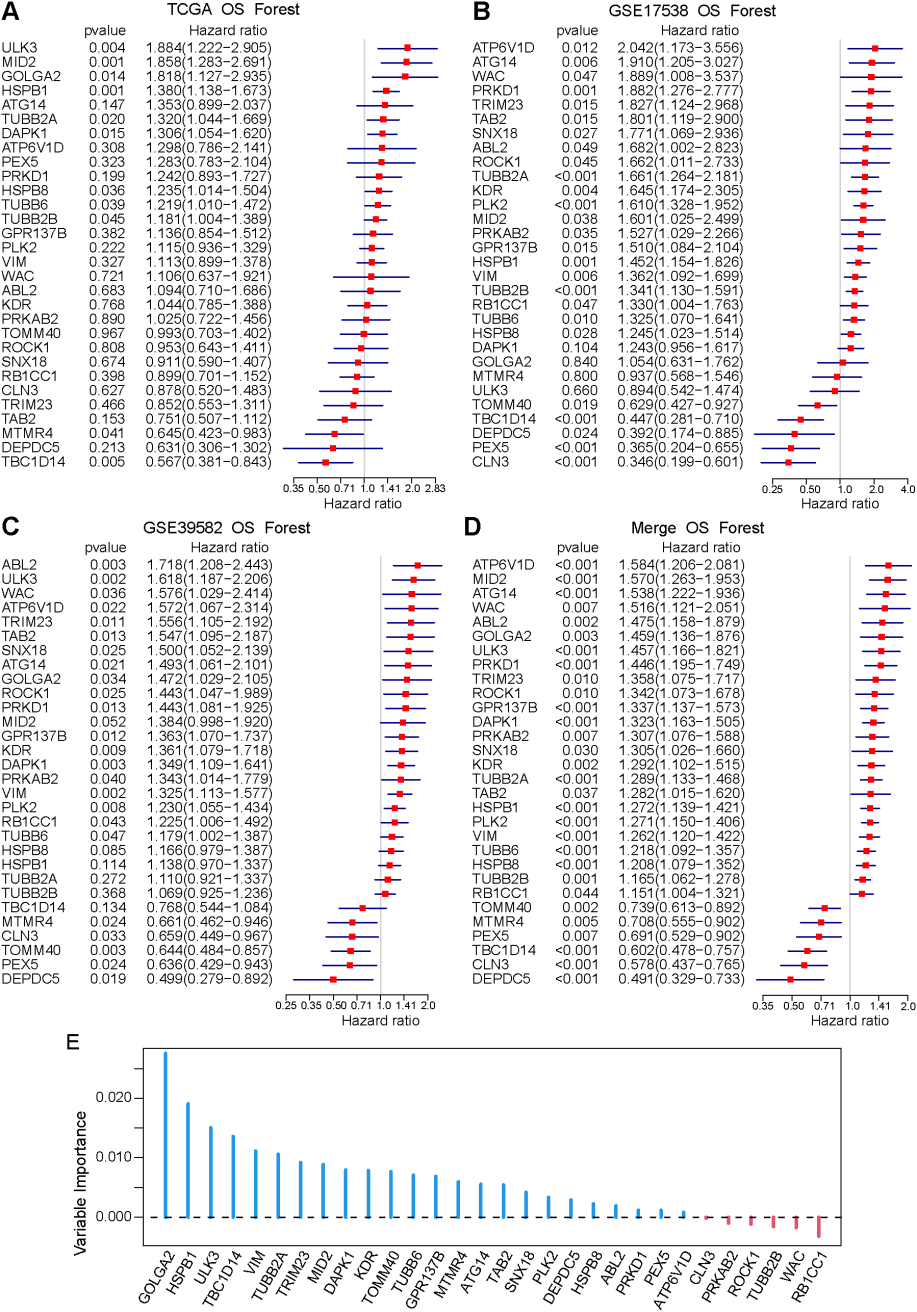
Construction of an autophagy-related prognostic signature using machine learning. **(A–D)** Univariate Cox regression analysis of autophagy-related genes across four datasets. A Venn diagram illustrates the overlap of genes with Cox P < 0.05.24 risk genes and 6 protective genes consistently significant in at least three datasets were identified; **(E)** Gene importance ranking derived from the random survival forest (RSF) model.

To develop and validate an autophagy-related prognostic signature in colorectal cancer, we conducted a comprehensive prognostic analysis across three independent datasets (TCGA, GSE17538, and GSE39582), as well as a merged cohort (TCGA + GSE17538 + GSE39582). The TCGA cohort was used for model training and internal cross-validation, whereas GSE17538 and GSE39582 were used as independent external validation cohorts. The merged cohort was used only for overall evaluation and visualization after cross-cohort batch correction, and it was not used for feature selection or model training. Univariate Cox regression analysis in the TCGA cohort identified 30 prognostic genes, comprising 24 risk-associated genes and 6 protective genes, all of which were significantly associated with overall survival (**Figures 3A–D**). To optimize predictive performance, we systematically evaluated ten classical machine-learning algorithms, including Random Survival Forest (RSF), LASSO, Gradient Boosting Machine (GBM), Survival-SVM, SuperPC, Ridge regression, plsRcox, CoxBoost, Stepwise Cox, and Elastic Net. Among these, RSF, LASSO, CoxBoost, and Stepwise Cox were further combined with other models due to their inherent dimensionality-reduction and feature-selection advantages. Among all algorithms, the RSF-based model exhibited the best prognostic performance, achieving a mean C-index of 0.72–0.73 (**Supplementary Figure 6**). Based on the optimal RSF model, we ranked the relative importance of the 30 prognostic genes, identifying GOLGA2, HSPB1, ULK3, TBC1D14, and VIM as the top five key genes contributors to the prognostic signature (**Figure 3E**).

### 3.4 Validation and multi-omics characterization of the RSF-based prognostic model

**Figure 4:**
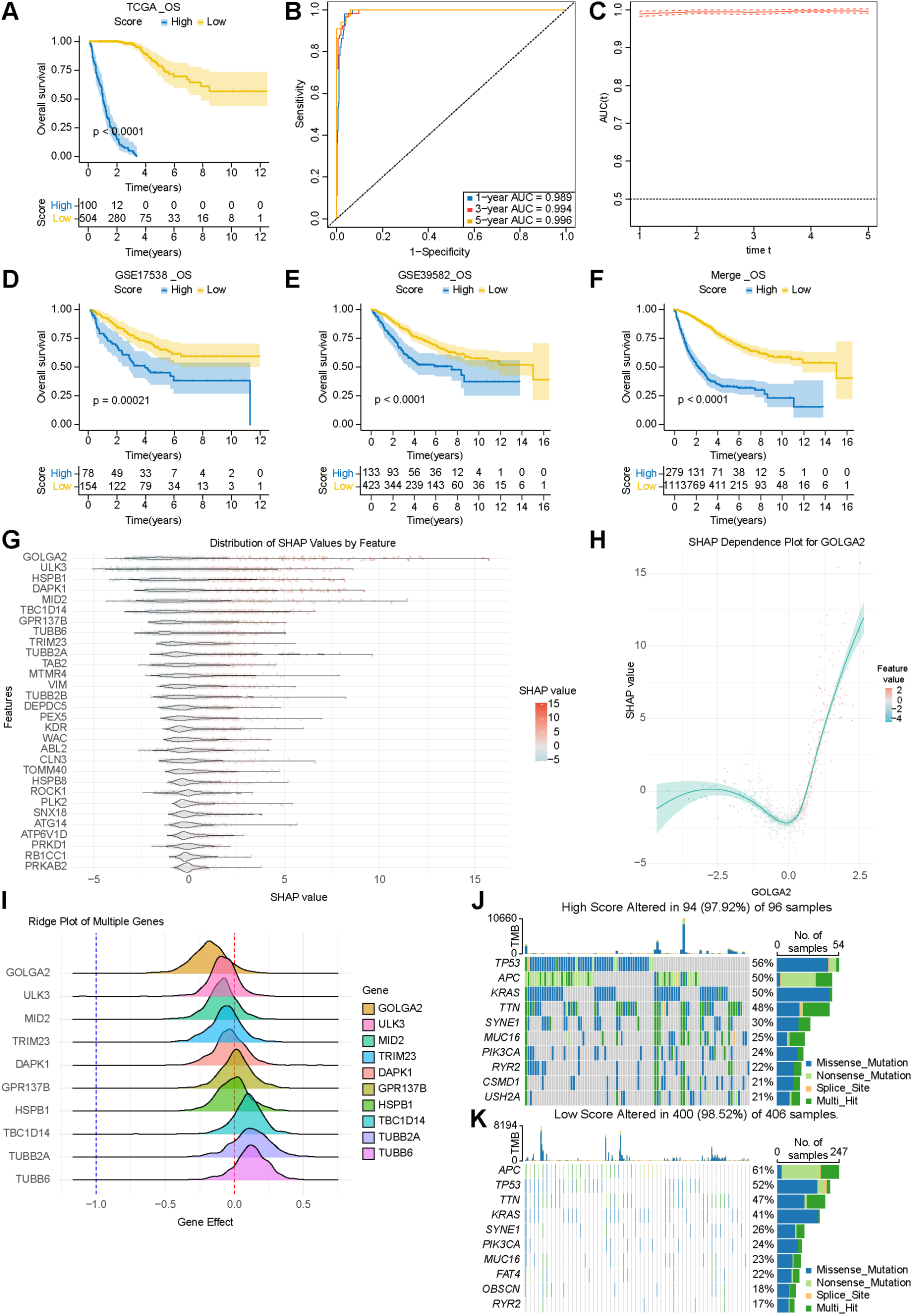
Development, validation, and interpretation of the RSF-based prognostic model. **(A)** Kaplan–Meier survival analysis of high- and low-risk groups in the TCGA training cohort; **(B)** Receiver operating characteristic (ROC) curve analysis of the RSF model in the training cohort (AUC > 0.98); **(C)** Time-dependent ROC curves for predicting 1-, 3-, and 5-year overall survival; **(D–F)** Kaplan–Meier survival analyses in two independent validation cohorts and a merged cohort (P < 0.01); **(G)** SHAP (SHapley Additive exPlanations) summary plot illustrating the contribution of individual genes to model predictions; **(H)** SHAP dependence plot for the top-ranked gene GOLGA2, showing the relationship between gene expression and SHAP values; **(I)** CRISPR dependency scores from the Cancer Dependency Map (DepMap) for the top 10 genes identified by SHAP analysis; ridge plots depict gene effect distributions, with more negative values indicating higher essentiality for cell survival; **(J, K)** Somatic mutation landscapes of the high-risk and low-risk groups generated using the maftools package.

Using the RSF-based prognostic model, patients in the TCGA training cohort were stratified into high- and low-risk groups with a highly significant difference in overall survival (P < 0.0001, **Figure 4A**). The model exhibited excellent predictive accuracy, with the areas under the receiver operating characteristic curve (AUCs) for 1-, 3-, and 5-year overall survival all exceeding 0.98 (**Figures 4B, C**). The very high AUC values observed in the training cohort may reflect stronger fitting to the training data. To evaluate the generalizability of the RSF-based signature, we further validated the RSF signature in two independent external cohorts (GSE17538 and GSE39582) as well as in a merged dataset. Consistently, the model stratified patients into distinct high- and low-risk groups across all validation cohorts, each showing significant survival differences (**Figures 4D–F**). Clinicopathological correlation analysis revealed that the high-risk patients were significantly enriched for advanced tumor stage and mortality (**Supplementary Figures 7A–D**), supporting the strong prognostic discrimination of the RSF model.

Single-cell analysis of the cell type-specific expression pattern of GOLGA2, a top-ranked autophagy-related gene, showed that GOLGA2 was broadly lowly expressed across most immune cell populations, while exhibiting relatively enriched expression in stromal compartments, particularly in myofibroblasts/fibroblasts (**Supplementary Figure 7E**). Moreover, subsets of plasma cells and mast cells also displayed elevated GOLGA2 expression, indicating substantial intrapopulation heterogeneity, and these cell populations were characterized by relatively high autophagy scores. Together, these results indicate that GOLGA2 displays a cell type-specific expression pattern in CRC, with preferential enrichment in stromal-related cell populations, and may be functionally linked to autophagy.

Furthermore, SHapley Additive exPlanations (SHAP) analysis was applied to quantify gene-level feature contributions and interpret the model (**Figure 4G**), identifying it as the most influential predictor (**Figure 4H**). CRISPR-Cas9 dependency data obtained from the Cancer Dependency Map showed that GOLGA2, MID2, TRIM23, and ULK3 are essential for colorectal cancer cell survival, as reflected by negative gene effect scores, whereas TBC1D14 may function as a growth suppressor (**Figure 4I**). Mutation spectrum profiling revealed a strong similarity between the high-risk group and Cluster D, and between the low-risk group and Clusters B/C (**Figures 4J, K**), indicating genomic concordance between the risk model and molecular subtypes. High-risk tumors inferred an immune-suppressive TME characterized by increased stromal and endothelial components and elevated infiltration of M2-like macrophages (**Supplementary Figure 8**).

These tumors also exhibited upregulation of multiple chemokine and cytokine signaling pathways—including CCL18, CCL17, CXCL12, EGF/EGFR, VEGF/VEGFR, PDGF, and TGF-β—indicating enhanced stromal remodeling and immunosuppression (**Supplementary Figure 9**). Gene enrichment analysis further demonstrated that high-risk tumors were associated with extracellular matrix reorganization and cell-cycle activation, whereas low-risk tumors were enriched for DNA repair and differentiation pathways (**Supplementary Figures 10A–E**). Together, these findings indicate that high-risk tumors display enhanced, invasive, and microenvironment-remodeling capabilities.

Drug sensitivity was explored by estimating IC50 values using oncoPredict. Predicted IC50 values differed between the high-risk and low-risk groups for multiple compounds. The top 9 compounds ranked by P value are shown in **Supplementary Figure 11**. Compared with the low-risk group, the high-risk group showed higher predicted IC50 values for several compounds, suggesting lower predicted sensitivity in this exploratory analysis.

### 3.5 Experimental validation of key autophagy-related prognostic genes

**Figure 5:**
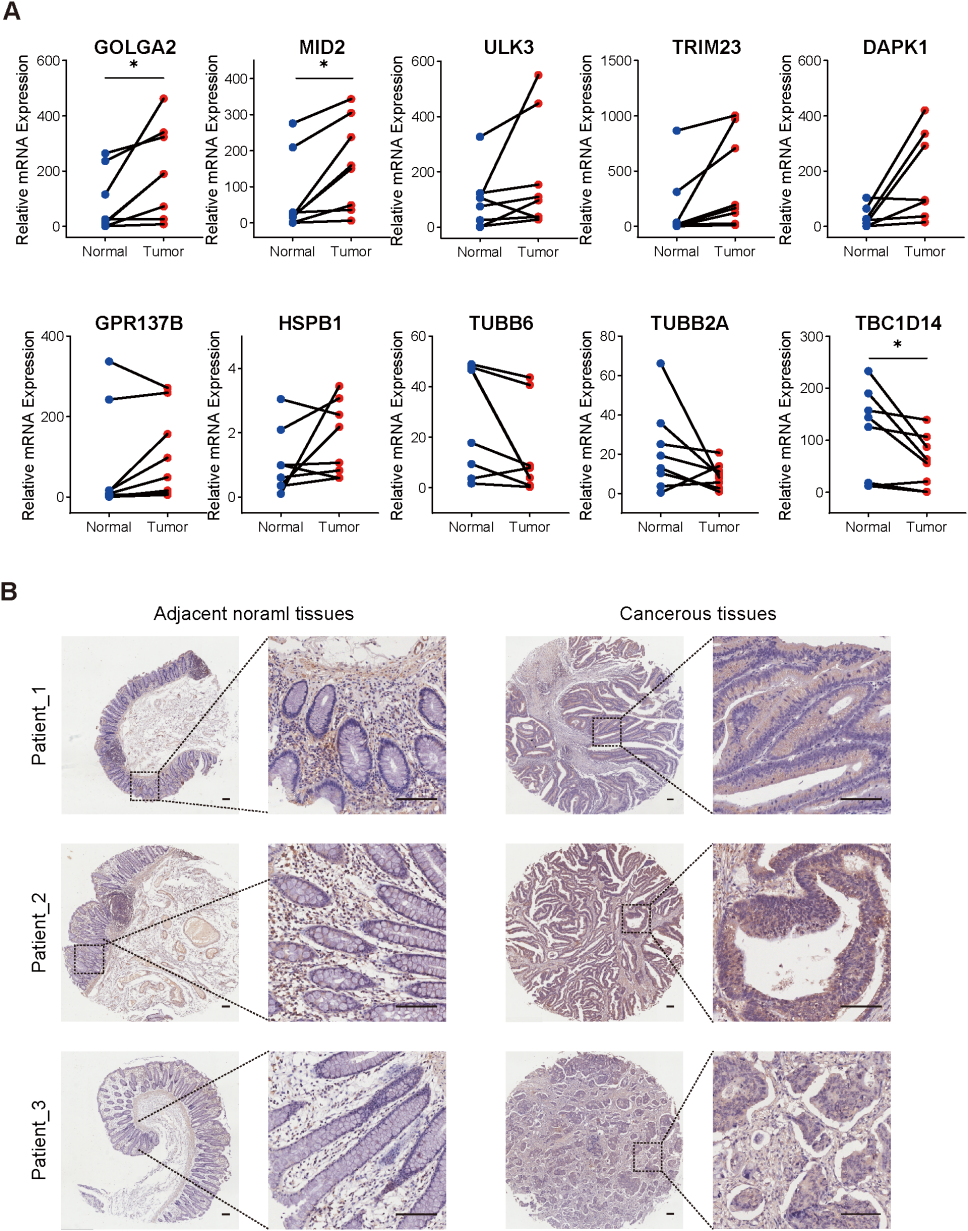
Experimental validation of key autophagy-related genes in clinical CRC samples. **(A)** Relative mRNA expression levels of 10 key genes from the prognostic model in paired colorectal cancer and adjacent normal tissues (n = 8), measured by qRT-PCR. GOLGA2 and MID2 were significantly upregulated, whereas TBC1D14 was downregulated in tumor tissues. **(B)** Representative immunohistochemistry (IHC) images showing GOLGA2 protein expression in CRC tissues and adjacent normal mucosa (n = 8). Scale bars: 100 μm. *P < 0.05.

Based on the SHAP-derived gene importance rankings from the RSF prognostic model, we performed experimental validation of the top 10 candidate genes (GOLGA2, ULK3, TUBB6, TUBB2A, TRIM23, TBC1D14, MID2, DAPK1, HSPB1, and GPR137B) in clinical colorectal cancer specimens. QRT-PCR analysis of eight paired tumor and adjacent normal tissues demonstrated significantly elevated mRNA expression levels of GOLGA2 and MID2 in tumor samples, whereas TBC1D14 was markedly downregulated (**Figure 5A**). Immunohistochemistry further confirmed substantially increased GOLGA2 protein expression in tumor tissues across eight patient samples (**Figure 5B**). Collectively, these results indicate that the genes identified by RSF model are functionally involved in colorectal carcinogenesis, with GOLGA2—consistently upregulated at both mRNA and protein levels—emerging as a putative oncogenic driver in CRC.

### 3.6 Functional characterization of GOLGA2 in HCT116 colorectal cancer cells

**Figure 6:**
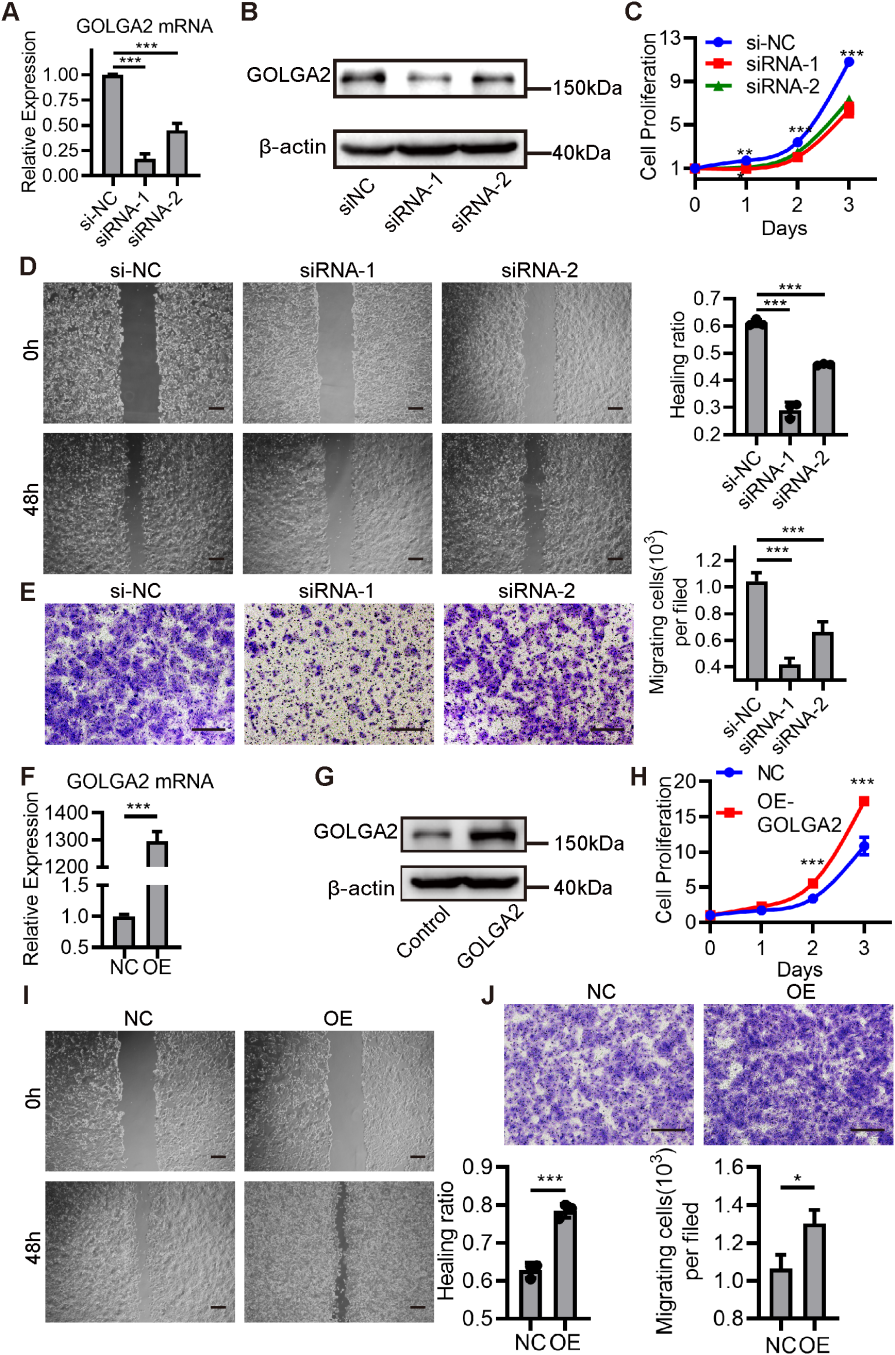
Functional validation of GOLGA2 in colorectal cancer cells. **(A)** qRT-PCR analysis of GOLGA2 mRNA levels in HCT116 cells transfected with negative control (NC) siRNA or GOLGA2-specific siRNAs. **(B)** Western blot analysis of GOLGA2 protein expression following siRNA-mediated knockdown. **(C–E)** Cell proliferation, wound healing, and Transwell migration assays showing that GOLGA2 knockdown significantly inhibited cell proliferation and migration. **(F)** qRT-PCR analysis of GOLGA2 mRNA levels following transfection with a GOLGA2 overexpression plasmid. **(G)** Western blot analysis confirming GOLGA2 overexpression. **(H–J)** Cell proliferation, wound healing, and Transwell migration assays demonstrating that GOLGA2 overexpression significantly enhanced proliferative and migratory capacities. Data are presented as mean ± SD. *P < 0.05, **P < 0.01, ***P < 0.001 (Student’s t-test).

To elucidate the functional role of the autophagy-associated gene GOLGA2 in CRC, we performed both loss- and gain-of-function assays in HCT116 colorectal cancer cells. Transfection with GOLGA2-specific siRNA efficiently reduced GOLGA2 mRNA and protein expression levels (**Figures 6A, B**). Functional assays demonstrated that GOLGA2 knockdown significantly suppressed cell proliferation (**Figure 6C**). Moreover, wound-healing and Transwell migration assays showed that GOLGA2 silencing markedly impaired cellular migratory capacity (**Figures 6D, E**). Conversely, enforced overexpression of GOLGA2 using a plasmid construct promoted cell proliferation (**Figures 6F, G**) and enhanced migration ability in both wound-healing and Transwell assays (**Figures 6H–J**).

## 4 Discussion

This study clarifies how autophagy relates to an immunosuppressive TME in CRC by using integrated multi-omics data and explainable machine-learning analysis. Our findings reveal heterogeneous autophagic activity across the TME that promotes a pro-tumor niche by strengthening tumor–stroma interactions while impairing immune activation. Importantly, the autophagy-based molecular subtyping and prognostic model not only provides a robust tool for outcome prediction but also identifies GOLGA2 as a key autophagy-related oncogenic driver in CRC.

Single-cell transcriptomic analysis revealed pronounced cell-type-specific autophagy patterns, with malignant and stromal cells exhibiting markedly elevated autophagic activity compared with lymphoid cells. This spatial heterogeneity indicates distinct functional specialization within the TME, consistent with recent reports describing cell-specific autophagy regulation in cancer ecosystems (18). Previous studies have shown that autophagy enhances metabolic plasticity in tumor cells, facilitates therapy resistance, and contributes to immune evasion by establishing an immunosuppressive niche (19, 20). Consistent with these findings, the high-autophagy subtype in our study displayed a “tumor-stroma dominant” phenotype enriched for epithelial–mesenchymal transition (EMT), hypoxia, and mTORC1 signaling pathways (21–23). Furthermore, cell–cell interaction analysis demonstrated that high autophagy is associated with strengthened tumor-stroma communication but attenuated immune–immune communication within the TME.

Cluster D—characterized by an immunosuppressive microenvironment and poor prognosis—displayed a high prevalence of mutations in TP53, APC, and KRAS. These clinical driver mutations reprogram cellular metabolism, impair protein homeostasis, and promote the accumulation of damaged organelles (24). APC mutations occur early in colorectal carcinogenesis, initiating aberrant Wnt/β-catenin activation and adenoma formation (25). Subsequent KRAS mutations facilitate malignant progression via constitutive MAPK and PI3K signaling (26, 27). Loss of TP53 further accelerates tumor progression by compromising genome stability and apoptotic responses (28, 29). Autophagy mitigates metabolic and oxidative stress induced by oncogenic KRAS signaling by recycling intracellular substrates and helps TP53-deficient cells withstand persistent stress and resist therapy, collectively promoting tumor evolution and metastatic dissemination (30, 31).

Cluster D also exhibited hallmark features of an immune-cold TME, including abundant regulatory T cells and M2 macrophages, but scarce cytotoxic CD8⁺ T cells. Although radiotherapy, chemotherapy, and targeted therapies can induce immunogenic cell death (ICD) and activate antitumor immunity (32), autophagy counteracts ICD by clearing damaged cellular components and reducing the release of danger-associated molecular patterns (DAMPs) (33). Additionally, autophagy can impair antigen presentation by degrading tumor antigens or MHC-I molecules (34), promote CD8⁺ T-cell exhaustion through persistent antigen exposure (35, 36), enhance Treg suppressive function, and drive macrophage polarization toward an M2 phenotype (37, 38). Autophagy also supports CAF-mediated extracellular matrix (ECM) remodeling, generating a dense stromal barrier that restricts T-cell infiltration (18). Together, these mechanisms illustrate how autophagy orchestrates the formation of an immune-refractory niche, explaining the adverse prognosis associated with Cluster D.

This work adds several points that were not clear before. It maps autophagy activity at single-cell resolution across major CRC TME compartments, instead of treating autophagy as one bulk signal. It links this cell-type pattern to immune features, including reduced immune communication and an immune-suppressive setting in the high-autophagy subtype. It also provides an autophagy-based molecular classification and a prognostic model that support patient risk stratification, and it connects the model to functional evidence through GOLGA2 validation. We also explored potential drug sensitivity differences using oncoPredict and estimated IC50 values. Several compounds showed different predicted IC50 values between the high-risk and low-risk groups. These results are hypothesis-generating. They reflect computational predictions rather than measured drug responses. They should not be used to guide clinical drug selection without validation. Follow-up work can test these candidates in 3D models, patient-derived systems, or clinical cohorts with treatment outcomes.

Our predictive model further identified GOLGA2 as the strongest autophagy-related risk gene, a finding confirmed by functional assays. GOLGA2, a structural component of the Golgi apparatus, has been shown to regulate Golgi morphology, autophagosome formation, and tumor progression (39–42). Overexpression of GOLGA2 disrupts autophagosome–lysosome fusion, leading to LC3B accumulation and impaired autophagic flux (43). Consistently, GOLGA2 knockdown in lung cancer induces autophagy and suppresses tumor growth (44). Based on these findings, we propose that under high autophagic stress, tumor cells upregulate GOLGA2 to restrain excessive autophagic flux, thereby preventing autophagy-induced cell death while preserving metabolic resources for proliferation and migration. These results suggest that GOLGA2 may affect autophagy dynamics, which could influence immune-related features in the TME. This idea needs direct autophagic flux assays and immune-focused experiments.

Despite the strengths of our integrative approach, this study has limitations. One limitation is the moderate sample size, which may limit power for some gene-level comparisons among the 10 machine-learning-derived autophagy signature genes. Another limitation is that we did not define the full molecular steps by which GOLGA2 regulates autophagic flux in CRC, including how it affects autophagosome–lysosome fusion in this disease context. Autophagic flux and autophagy-dependent cell death were not directly tested in this study. Functional validation was performed only in HCT116 cells, so the findings may not represent all CRC models. In addition, our functional validation relied on 2D culture, which may not fully capture tumor architecture and microenvironment constraints. 3D models and *in vivo* studies are needed to test whether the same effects hold in a more physiological setting. We also lack *in vivo* validation, so the GOLGA2-related autophagy effects and their immune impact still need testing in animal models. Future work should employ spatial transcriptomics and multiplex imaging to map the spatial organization of autophagy-active cells within the TME, dissect context-specific autophagy–immune interactions, and investigate the generalizability of these mechanisms across other malignancies.

## 5 Conclusions

This study establishes autophagy as a central regulator of the immunosuppressive tumor microenvironment in colorectal cancer. Through integrated multi-omics and machine-learning analyses, we developed an autophagy-based classification and prognostic model that enables accurate patient stratification and identified GOLGA2 as a top-ranked autophagy-related gene within the signature. Together, these findings provide insight into how autophagy-related programs relate to the CRC microenvironment and may support prognostic assessment. Further spatial and *in vivo* studies are needed to confirm the microenvironment features and to test the underlying mechanisms.

## Data Availability

The original contributions presented in the study are included in the article/**Supplementary Material**. Further inquiries can be directed to the corresponding authors.
