# Supplementary material for: Integrated single-cell and bulk transcriptomics identify autophagy-related immune-suppressive subtypes and a prognostic signature in colorectal cancer
Source: Front Immunol. 2026 Apr 21;17:1820929. doi: 10.3389/fimmu.2026.1820929 (PMC13139068; doi:10.3389/fimmu.2026.1820929)
Supplement: Supplementary file 1 [file DataSheet1.docx]

**Supplementary Materials**

**Supplementary Table**

**Supplementary Table 1** siRNA used in this study

| Name | Sense | Antisense |
| --- | --- | --- |
| GOLGA2-siRNA-1 | CAAUAUCACGAUAGAGAAA | UUUCUCUAUCGUGAUAUUG |
| GOLGA2-siRNA-2 | GGACAAUGCUGCUACUCUA | UAGAGUAGCAGCAUUGUCC |

**Supplementary Table 2** Primers used in this study

| Gene | Forward (5' to 3') | Reverse (5' to 3') |
| --- | --- | --- |
| GOLGA2 | CCCGCGATGTCGGAAGAAA | GCATTGTCCTTGGGTGTATCCT |
| ULK3 | GAAGGACACTCGTGAAGTGGT | ACAATGTGGGGATGTCGAATG |
| TUBB6 | TGGTGGACTTAGAGCCAGG | CCCTTTCGCCCAGTTGTTC |
| TUBB2A | AATGAGGCTGCTGGTAACAAA | AAGGGTCCAGACCTGACAGA |
| TRIM23 | TGGTTGTAAACAAGCTCGGAG | ACTCTAGCACCTTCACTACAGC |
| TBC1D14 | TGGTGGTTCAGGCCAAAAAG | GAGCACAGCGTTTCCAATGC |
| MID2 | CCAGCCTCCGTGGTTCTTAAT | ACAGGTCAATTCAGACTCCAGT |
| DAPK1 | ACGTGGATGATTACTACGACACC | TGCTTTTCTCACGGCATTTCT |
| HSPB1 | ACGGTCAAGACCAAGGATGG | AGCGTGTATTTCCGCGTGA |
| GPR137B | CTTGTACTTCACGCAGGTGAT | CCAATTTCCCGTCTTTACCAGC |

**Supplementary Figure**

**
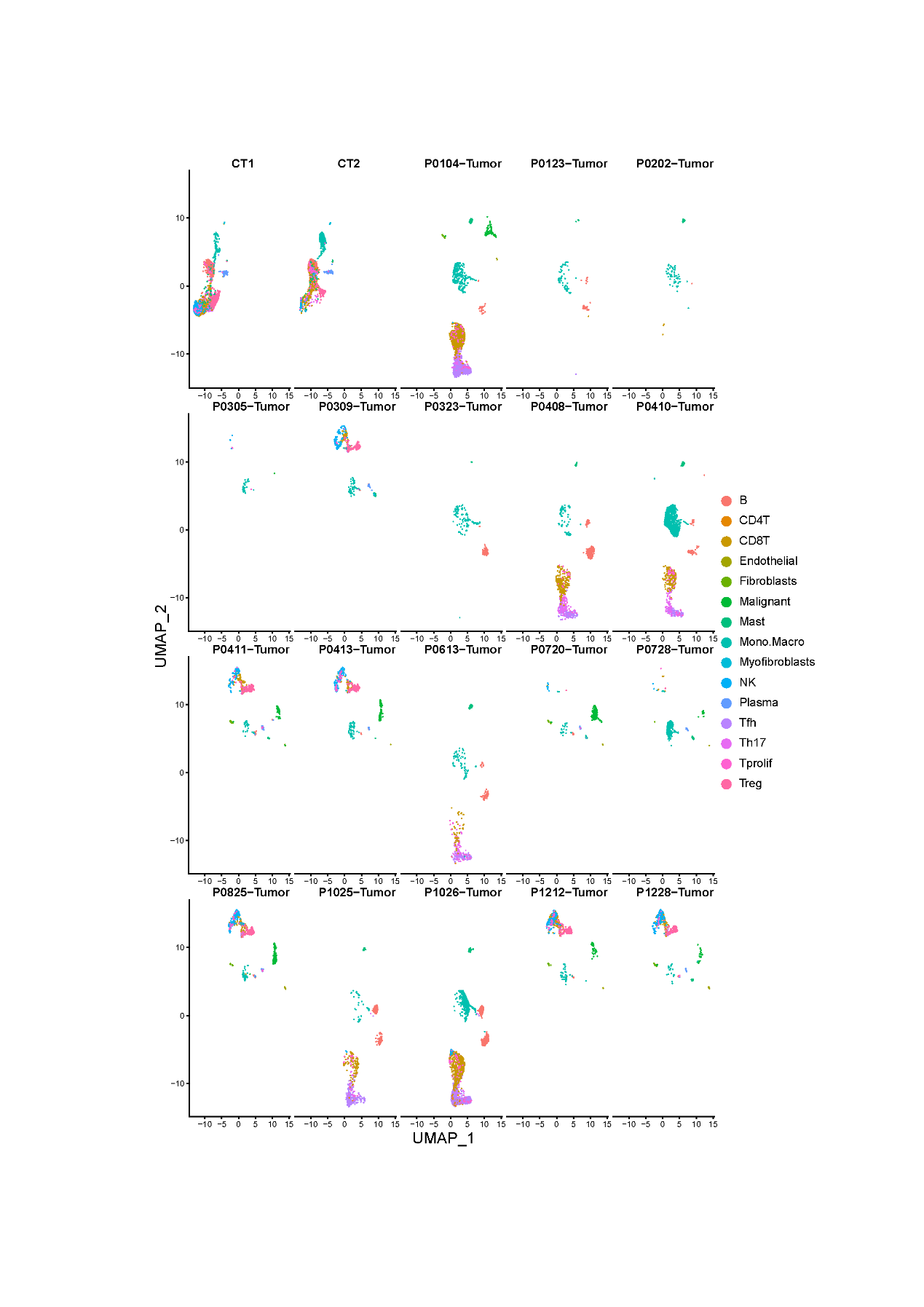
**

**Supplementary Figure 1.** Single-cell UMAP landscape of tumor cells from colorectal cancer patients UMAP visualization of tumor cells derived from single-cell RNA sequencing data of 20 colorectal cancer patients.

**
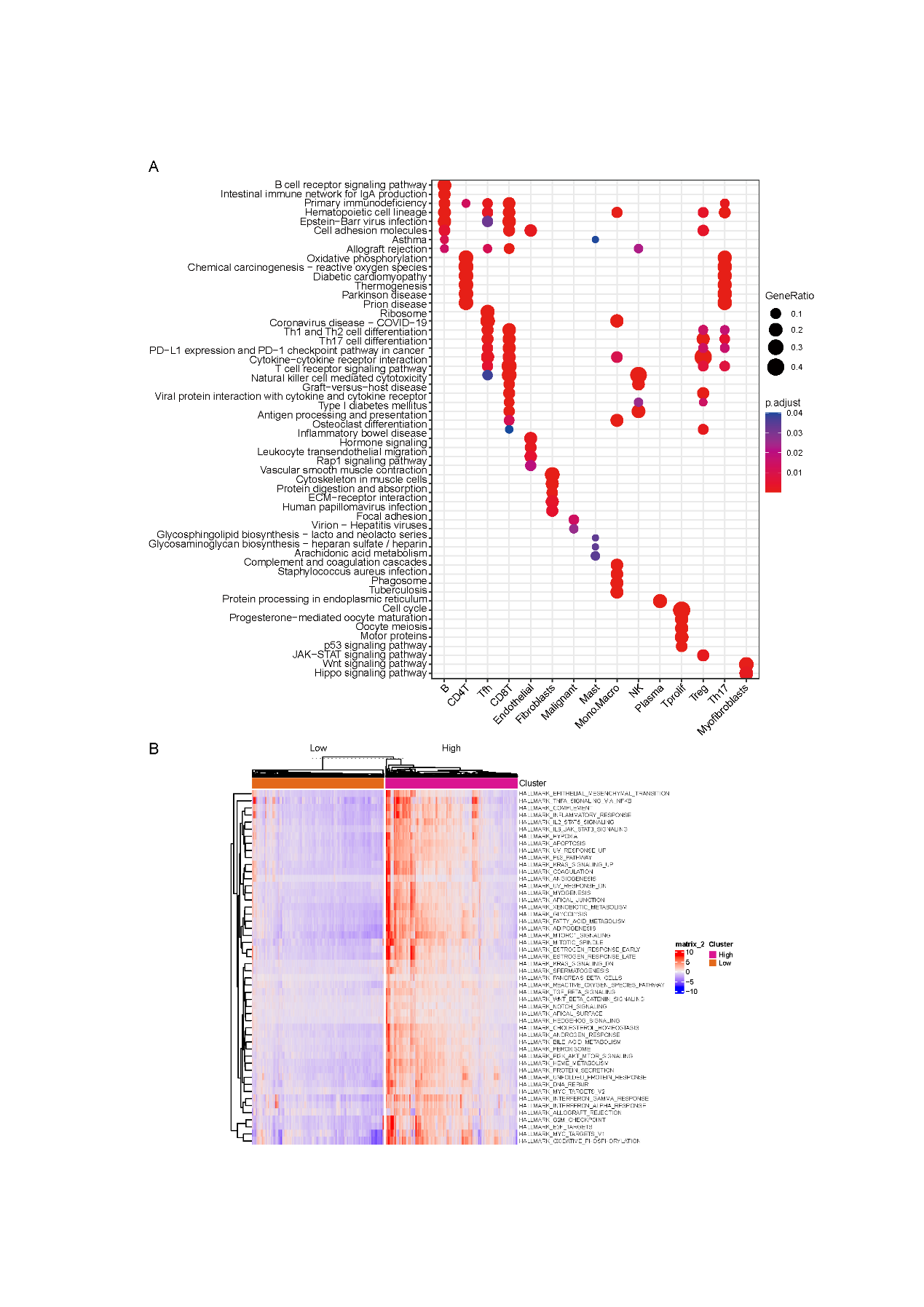
**

**Supplementary Figure 2.** Pathway enrichment analysis associated with cell types and autophagy activity (A) KEGG pathway enrichment analysis of the top 100 marker genes for each cell type identified by COSG analysis; (B) Heatmap showing enrichment scores of 50 Hallmark pathways obtained from the MSigDB database, comparing high- and low-autophagy groups stratified based on autophagy scores.

**
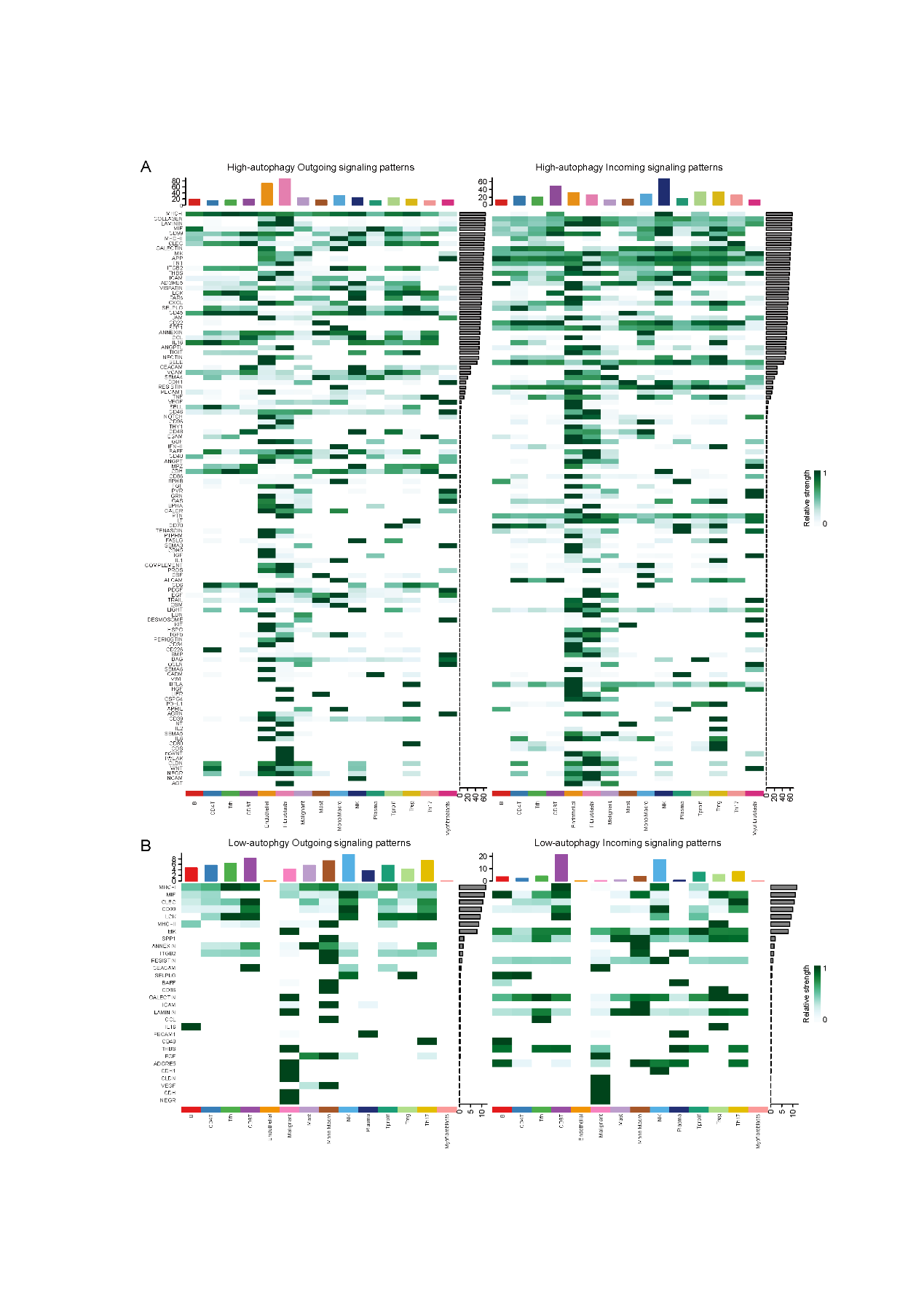
**

**Supplementary Figure 3.** Cell–cell communication patterns under different autophagy states (A) Incoming and outgoing signaling patterns and relative signal strengths among different cell types in the high-autophagy group, inferred from single-cell communication analysis; (B) Incoming and outgoing signaling patterns and relative signal strengths among different cell types in the low-autophagy group.

**
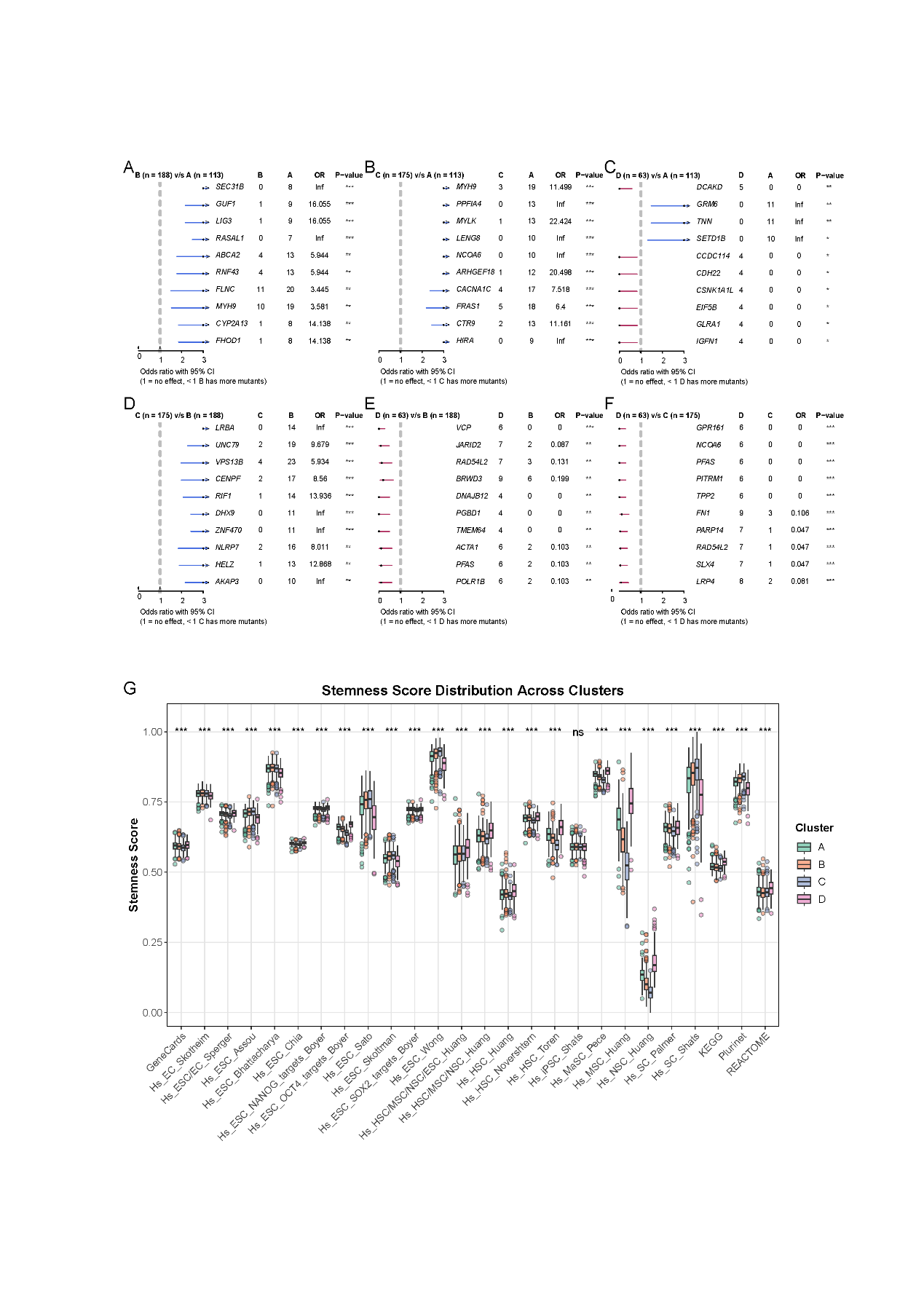
**

**Supplementary Figure 4.** Differential gene expression and stemness characteristics across autophagy-related clusters (A–F) Differentially expressed genes among different autophagy-related clusters; (G) Differences in cellular stemness scores among the four clusters.

**
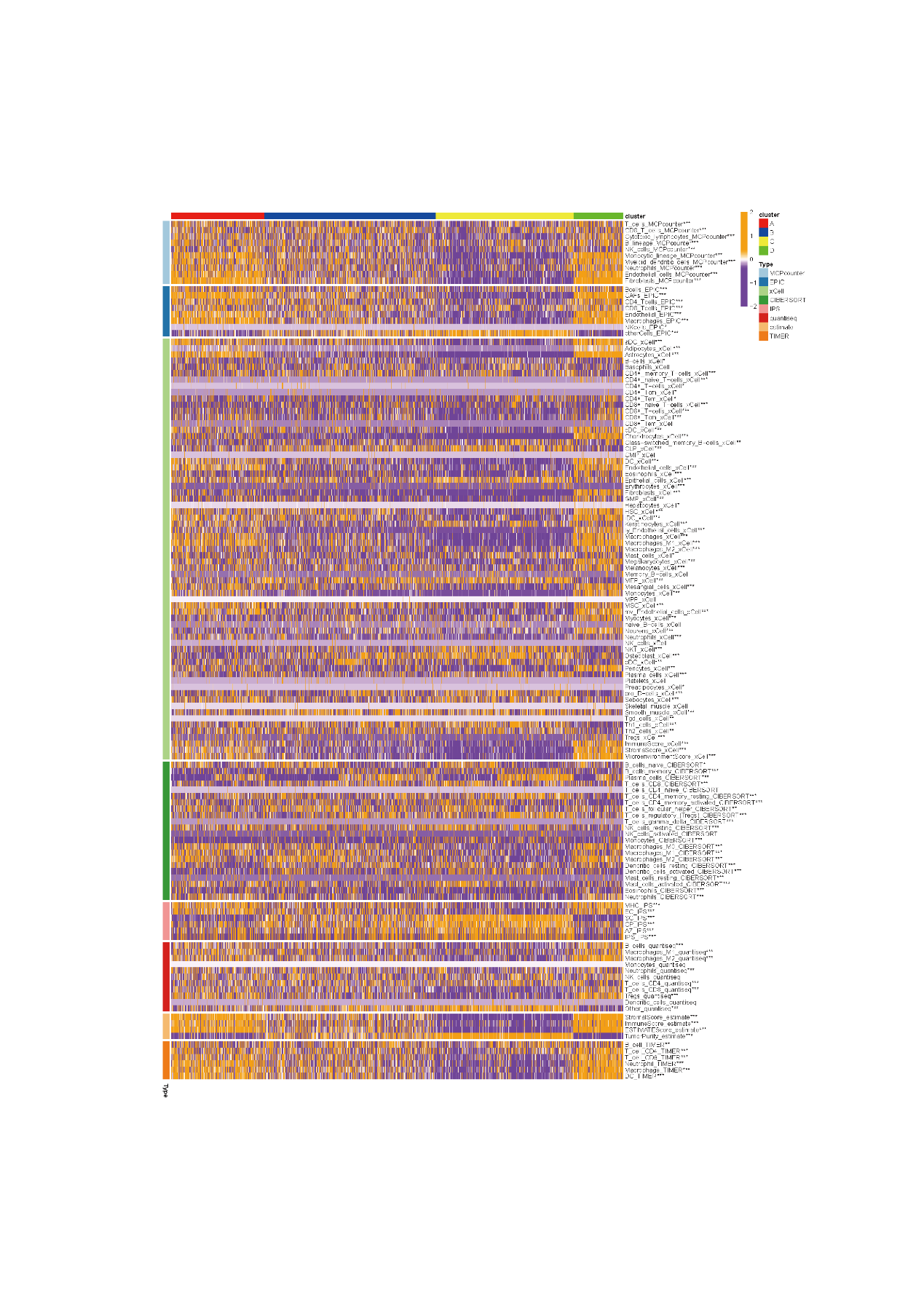
**

**Supplementary Figure 5.** Association between molecular subtypes and immune cell infiltration Heatmap illustrating the relationship between autophagy-related molecular subtypes and immune cell infiltration in the tumor microenvironment.


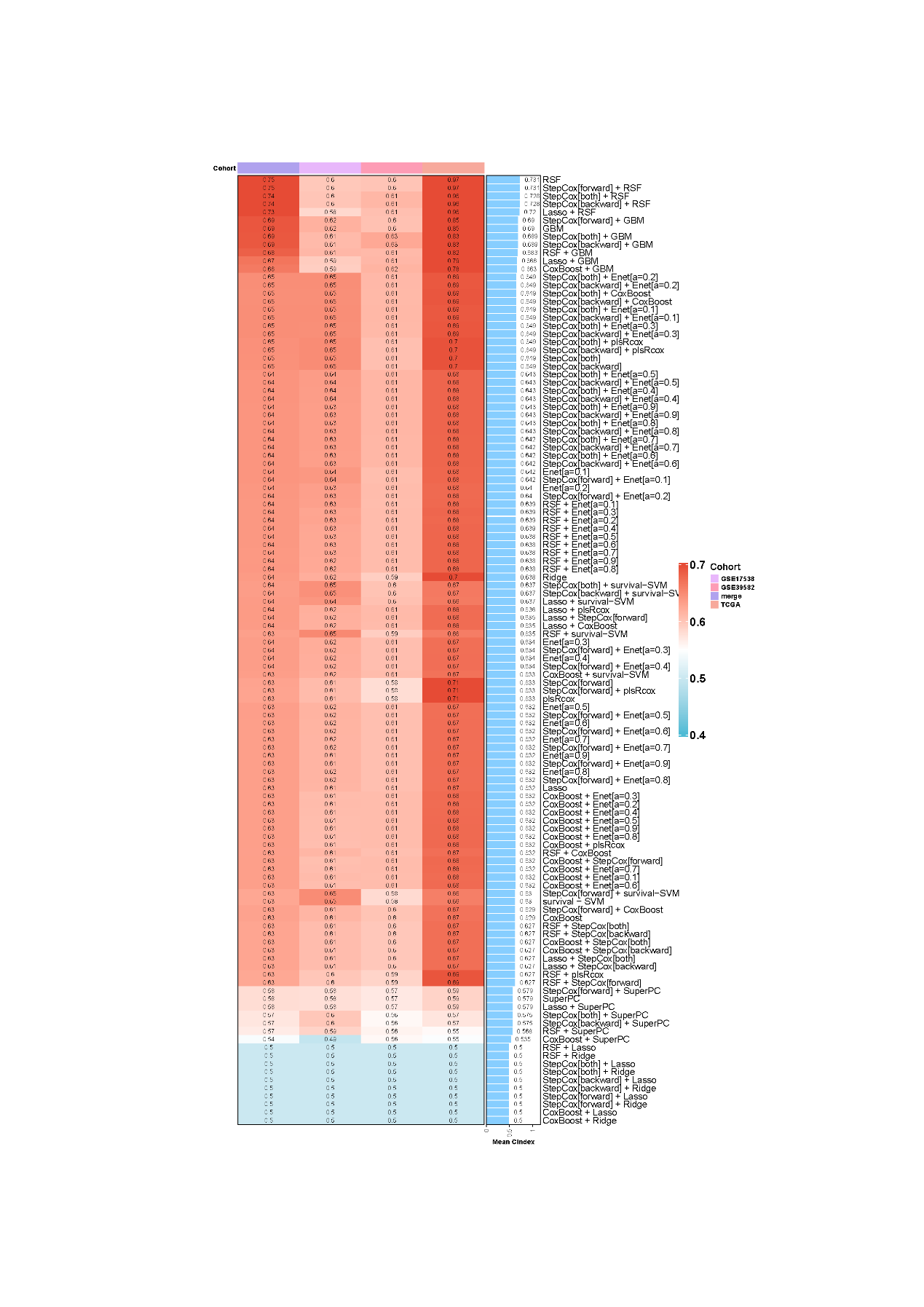


**Supplementary Figure 6.** Comparison of prognostic model performance across multiple machine learning strategies Concordance indices (C-indexes) of prognostic models constructed using ten machine learning algorithms and multiple algorithm combinations.


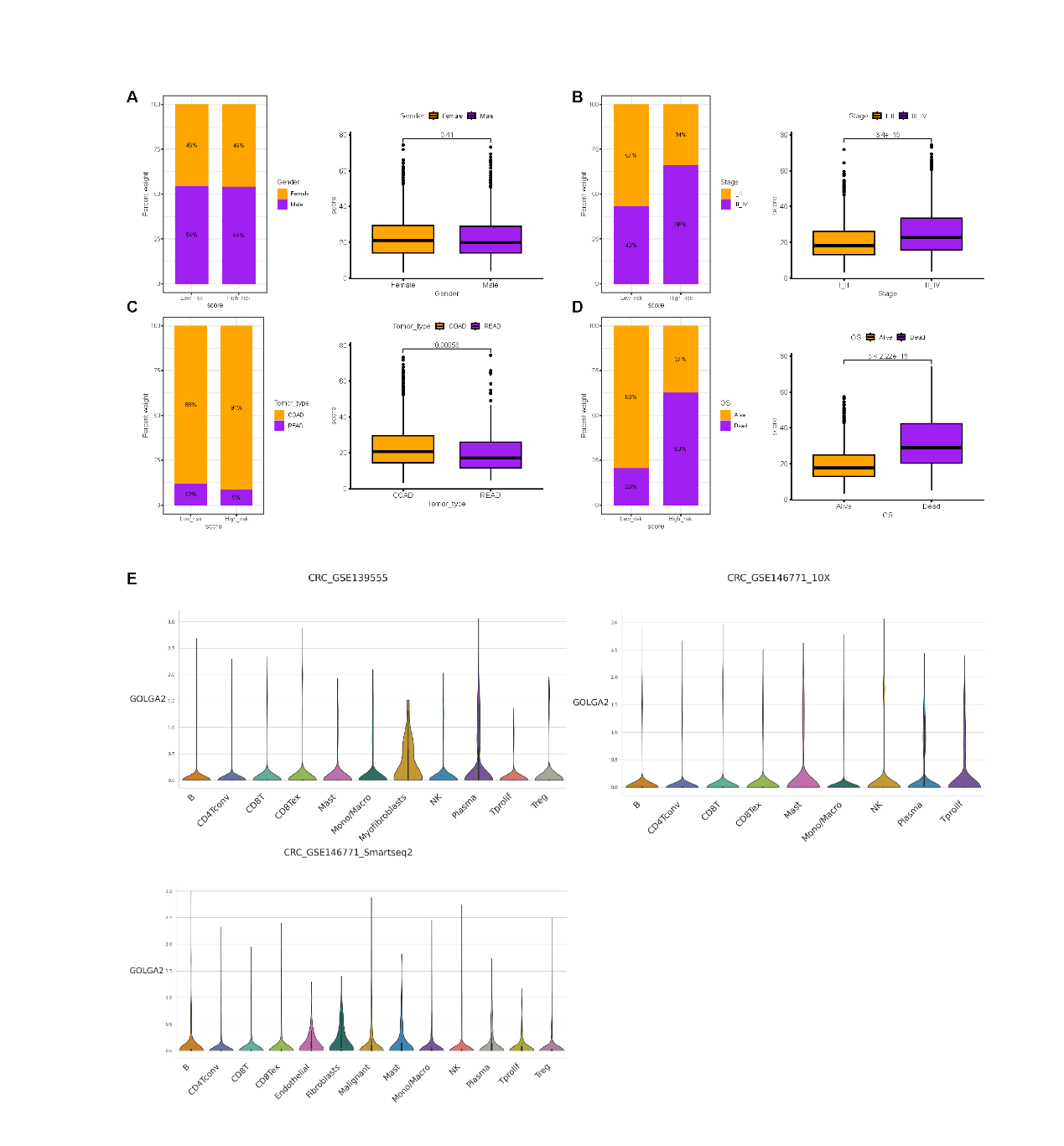


**Supplementary Figure 7.** Association between risk score and clinicopathological characteristics (A) Association between the prognostic model risk score and patient sex; (B) Association between risk score and tumor stage; (C) Association between risk score and tumor subtype; (D) Association between risk score and survival status; (E) (F) Violin plots show the distribution of GOLGA2 expression across annotated cell populations in CRC_GSE139555, CRC_GSE146771_10X, and CRC_GSE146771_Smartseq2 from the TISCH2 platform.

**
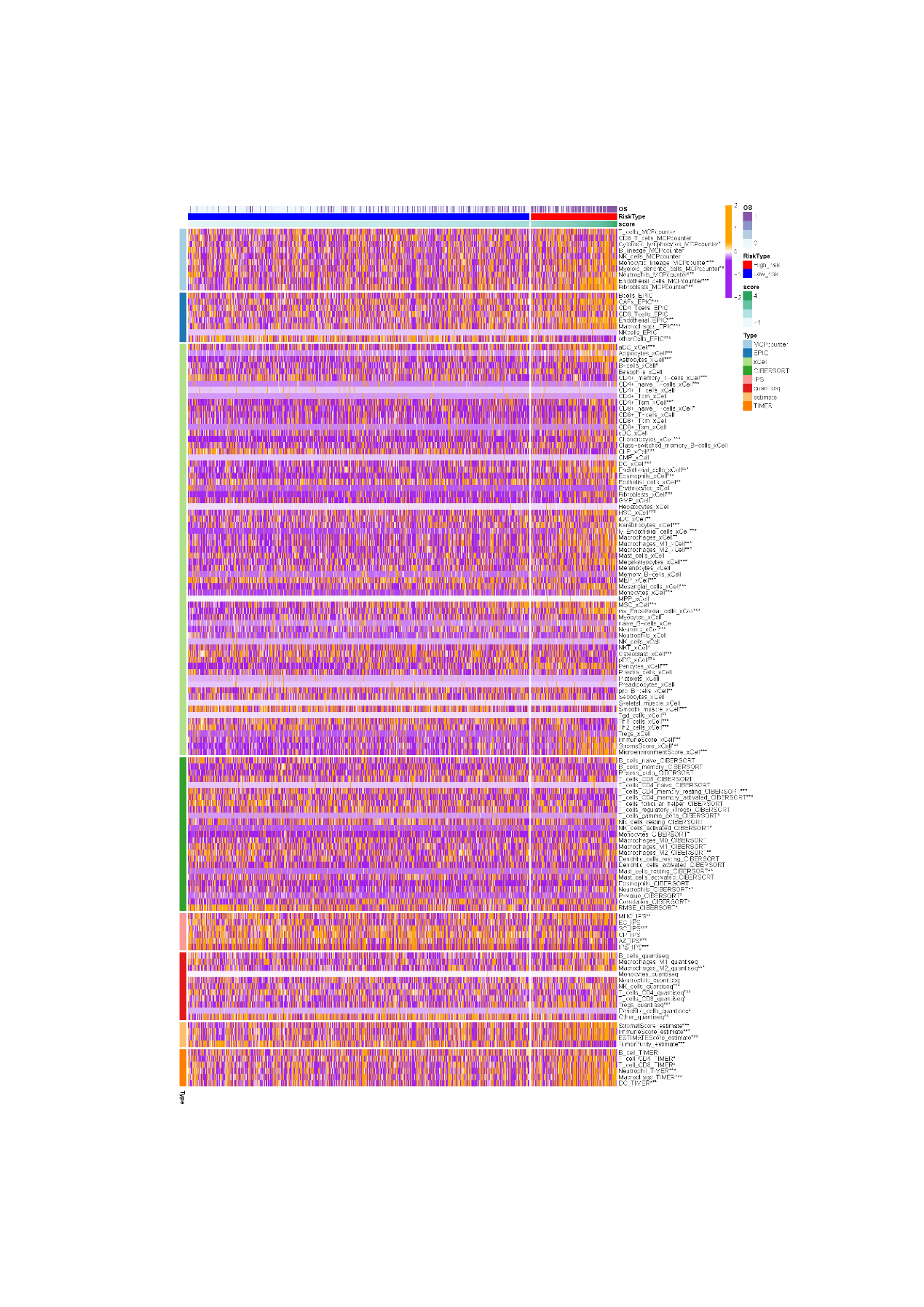
**

**Supplementary Figure 8.** Correlation between prognostic model risk score and immune cell infiltration Heatmap showing the relationship between the prognostic model risk score and the abundance of immune cell populations.

**
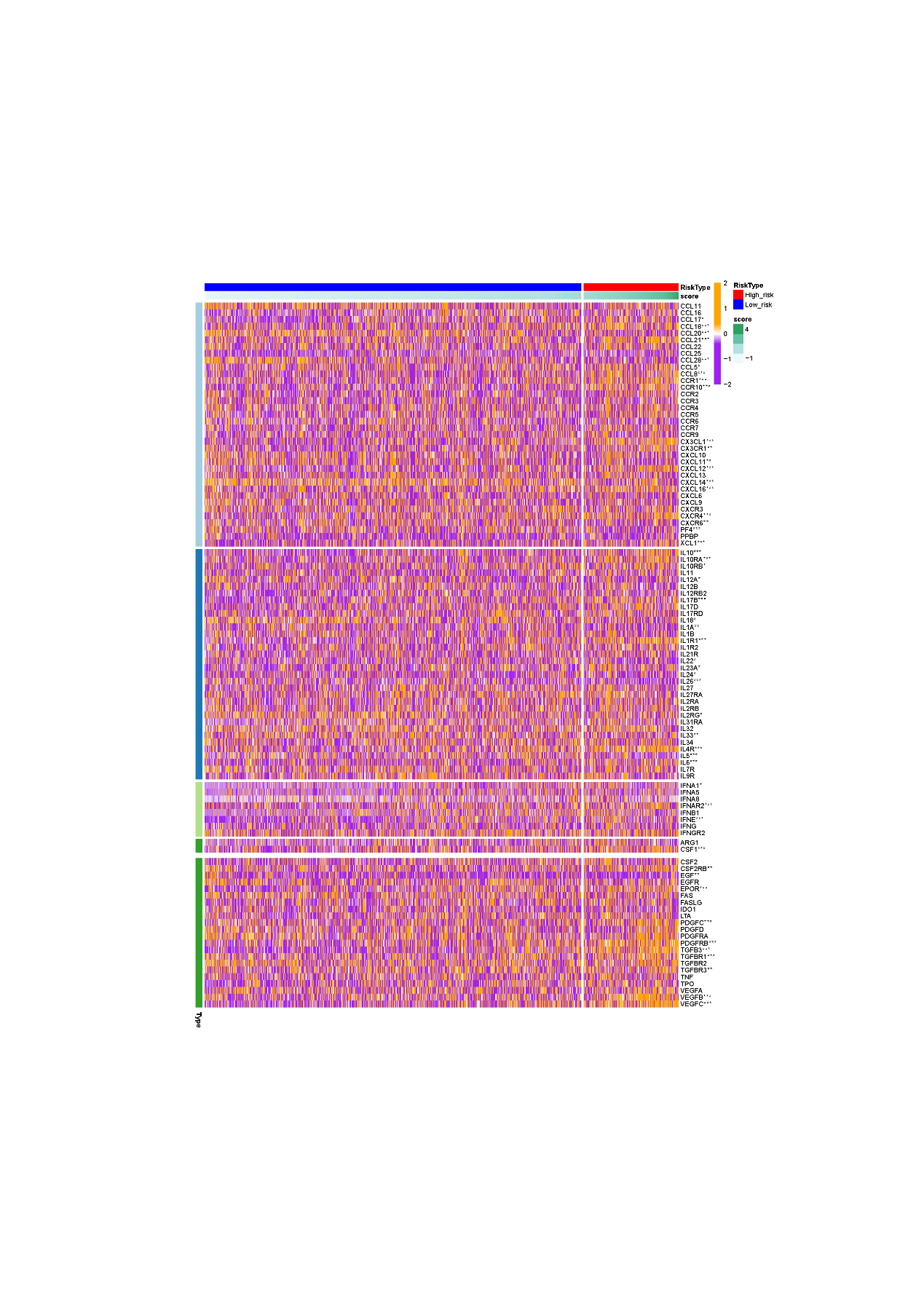
**

**Supplementary Figure 9.** Correlation between prognostic model risk score and chemokines and their receptors Heatmap illustrating correlations between the prognostic model risk score and the expression levels of chemokines and their corresponding receptors.

**
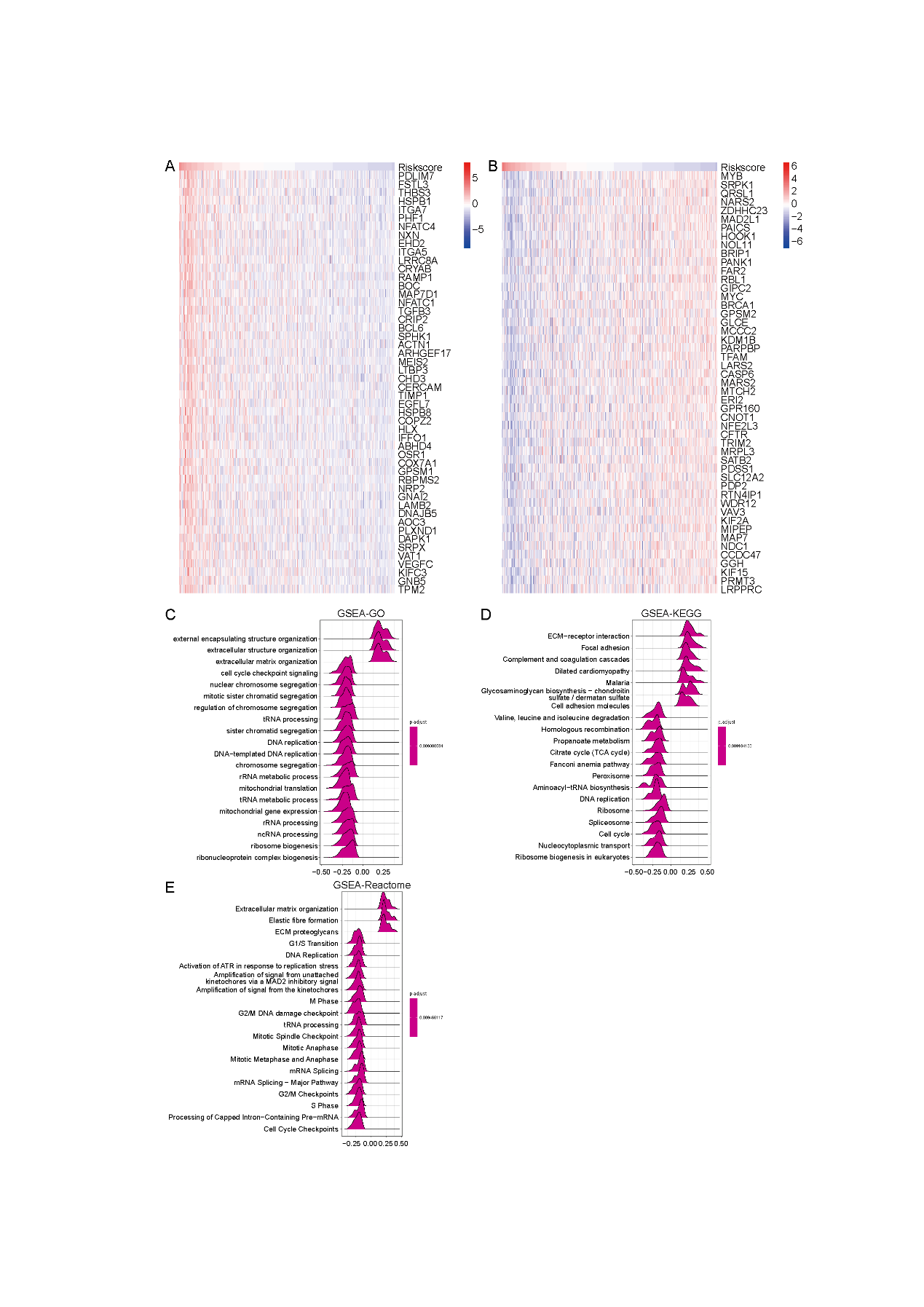
**

**Supplementary Figure 10.** Functional enrichment analysis associated with the RSF-derived risk score (A, B) Correlation analysis between the prognostic model risk score calculated by the random survival forest (RSF) model and all genes. Heatmaps display the top 50 positively correlated genes (A) and top 50 negatively correlated genes (B); (C–E) Gene set enrichment analysis (GSEA) based on the correlation results, including GO biological processes (C), KEGG pathways (D), and Reactome pathways (E), performed using the clusterProfiler R package.


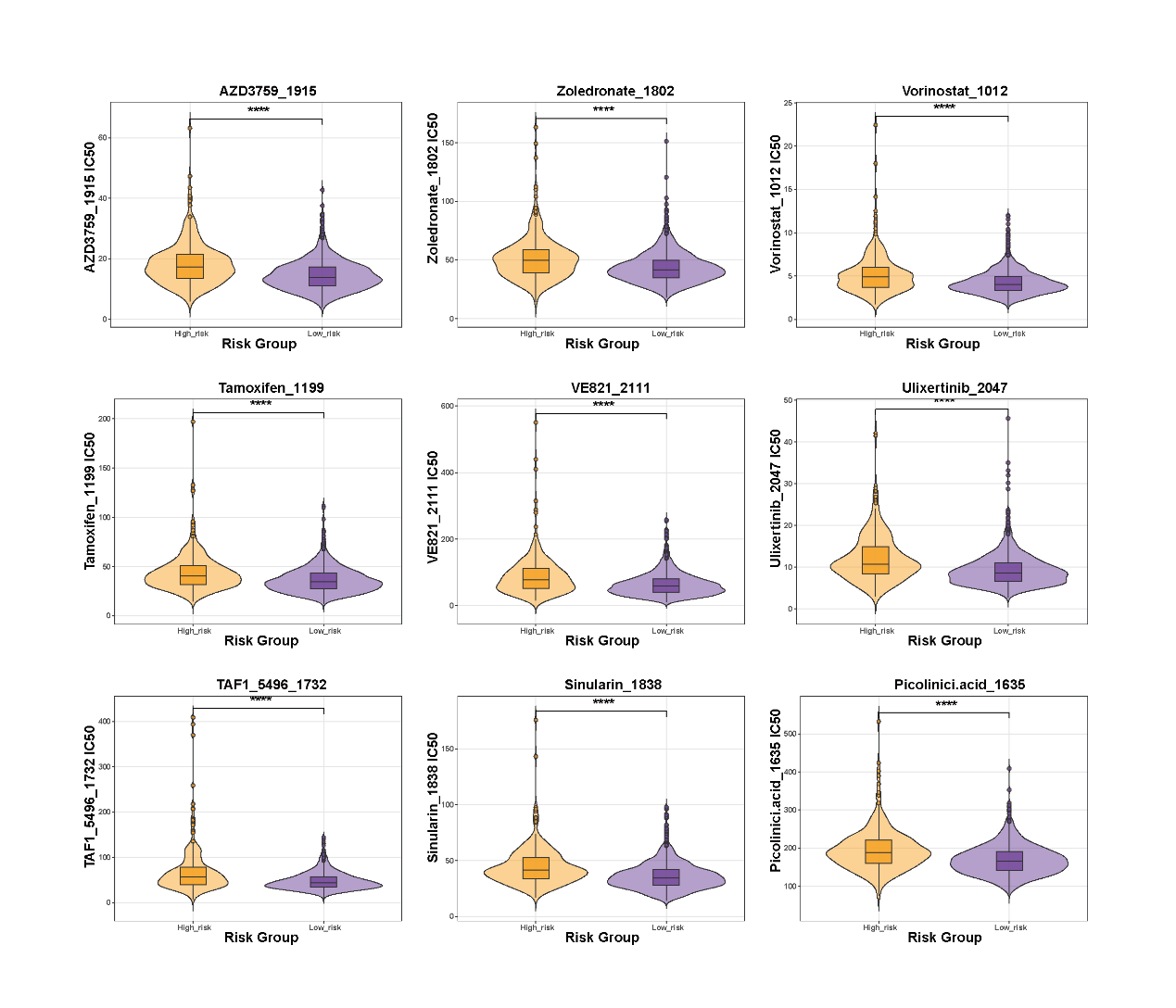


**Supplementary Figure 1**1. The half-maximal inhibitory concentration (IC₅₀) of multiple anticancer drugs for each sample was predicted using the R package oncoPredict, and differences in IC₅₀ values were compared between the high- and low-risk groups. Higher IC₅₀ indicates lower sensitivity to treatment (the 9 plots with the smallest p-values are shown).
